# Cytotoxic and Apoptotic Activities of *Prunus spinosa* Trigno Ecotype Extract on Human Cancer Cells

**DOI:** 10.3390/molecules22091578

**Published:** 2017-09-20

**Authors:** Stefania Meschini, Evelin Pellegrini, Maria Condello, Giovanni Occhionero, Sebastiano Delfine, Giancarlo Condello, Franco Mastrodonato

**Affiliations:** 1 National Center for Drug Research and Evaluation, Italian National Institute of Health, Rome 00161, Italy; evelin.pellegrini@guest.iss.it (E.P.); maria.condello@iss.it (M.C.); 2 Italian Society of Biointegrated Medicine, Bagnoli del Trigno, Isernia 86091, Italy; giovanniocchionero@hotmail.com (G.O.); mastrodonato.f@gmail.com (F.M.); 3 Department of Agriculture, Environment and Food Science, University of Molise, Campobasso 86100, Italy; delfine@unimol.it; 4 Department of Movement, Human and Health Sciences, University of Rome Foro Italico, Rome 00135, Italy; giancarlo.condello@gmail.com

**Keywords:** *Prunus spinosa*, Trigno ecotype, anticancer activity, apoptosis, mitochondrial dysfunction

## Abstract

The aim of this work was to demonstrate that a natural compound, not-toxic to normal cells, has cytotoxic and sensitizing effects on carcinoma cells, with the final goal of combining it with chemotherapeutic drugs to reduce the overall dose. *Prunus spinosa* Trigno ecotype (PsT) drupe extract with a nutraceutical activator complex (NAC) made of amino acids, vitamins and mineral salt blends, has shown in vitro anticancer activity. The cytotoxic effect of (PsT + NAC)^®^ has been evaluated on human cancer cells, with an initial screening with colorectal, uterine cervical, and bronchoalveolar cells, and a subsequent focus on colon carcinoma cells HCT116 and SW480. The viability reduction of HCT116 and SW480 after treatment with (PsT 10 mg/mL + NAC)^®^ was about 40% (*p* < 0.05), compared to control cells. The cell’s survival reduction was ineffective when the drug vehicle (NAC) was replaced with a phosphate buffer saline (PBS) or physiological solution (PS). The flow cytometry evaluation of cancer cells’ mitochondrial membrane potential showed an increase of 20% depolarized mitochondria. Cell cycle analysis showed a sub G1 (Gap 1 phase) peak appearance (HCT116: 35.1%; SW480: 11.6%), indicating apoptotic cell death induction that was confirmed by Annexin V assay (HCT116: 86%; SW480: 96%). Normal cells were not altered by (PsT + NAC)^®^ treatments.

## 1. Introduction

Nutritional and pharmacological factors may play a role in the prevention and treatment of several diseases [1]. The discovery of the anticancer properties of a natural diet supplement can be used to improve the chemotherapy efficacy and thus reduce the side effects of high dose therapy. Recent studies have proved the in vitro potential anticancer activity of plants and wild fruits [2,3,4]. The interest in plant phenolic extracts derives from the evidence of their potent antioxidant activity and their wide range of pharmacological properties, including anticancer activity. This led to our hypothesis of *Prunus spinosa* as an interesting compound.

*Prunus spinosa* Trigno ecotype (PsT) drupe extract with a nutraceutical activator complex (NAC) made of amino acids, vitamins and mineral salt blends, has been chemically prepared for evaluating the drug mechanisms of action at cellular levels. The aim of this work is to show that (PsT + NAC)^®^ is cytotoxic for cancer cells but non-toxic for normal cells and to identify the intracellular mechanisms involved in the cytotoxic behavior.

*Prunus spinosa* L. (blackthorn) belongs to the rose family (Rosaceae). It is a perennial deciduous plant growing as a shrub on wild uncultivated areas; although native of Italy, it can be also found in other European countries and in temperate regions of Asia. Despite being widespread in Italy, its ethnobotanical use is not well known as in other countries, where branch infusions are used in the treatment of hypertension and its macerated fruits for gastrointestinal disturbances [5]. The active compounds of *Prunus spinosa* mainly contain phenolic acids, flavonoids and anthocyanins [6]. Phenolic compounds are common constituents of fruits and vegetables and are considered an important class of antioxidant natural substances [6,7]. The remarkable diversity of their structures is the reason for their biological properties, such as bioavailability, antioxidant activity, specific interactions with cell receptors and enzymes [8]. Flavonoids have been reported to exert many biological activities in mammals, such as antibacterial, antiviral, analgesic, anti-allergic, hepatoprotective, cytostatic, apoptotic, estrogen and anti-estrogen functions [9,10]. Anthocyanins, from the flavonoids family, are found mainly in berries and have high antioxidant activity, which plays a vital role in the prevention of neuronal and cardiovascular illnesses, diabetes and cancer [11]. The present work is the first study dealing with the cytotoxic and apoptotic effects of a modified extract of *Prunus spinosa*. This study will show the antitumor activity of the PsT Trigno ecotype extract, supplemented with NAC, that has been patented by us [12].

## 2. Results

### 2.1. Chemical Composition of Prunus spinosa Trigno Ecotype Extract Performed by HPLC-DAD-ESI/MS Analysis

Plant extract was obtained from fresh parts of PsT drupes. The identification of active compounds such as phenolic acids, flavonoids and anthocyanins was done by means of liquid chromatography (HPLC) coupled with mass spectrometry (MS) using the electrospray ionization interface (ESI). In particular, among the phenolic acids—with a total of 38.36 mg/100 g—were, in higher quantities (see Table 1), the 3-*O*-Caffeoylquinic acid (26.31 mg/100 g) and the 4-*O*-Caffeoylquinic acid (8.97 mg/100 g). Among the flavonoids—with a total of 64.62 mg/100 g—were, Quercetin 3-*O*-rutinoside (21.98 mg/100 g) and Quercetin 3-*O*-glucoside (8.92 mg/100 g), others were quercetin and Kaempferol 3-*O*-rutinoside (6.22 mg/100 g). The anthocyanins—with a total of 0.63 μg/100 g—were, Cyanidin 3-*O*-rutinoside (42.31 µg/100 g), Cyanidin 3-*O*-glucoside (24.25 µg/100 g), Peonidin 3-*O*-rutinoside (15.53 µg/100 g) and Peonidin 3-*O*-glucoside (13.91 µg/100 g).

### 2.2. The Effect of (PsT + NAC)^®^ on the Human Cancer Cell Viability

**Figure 1:**
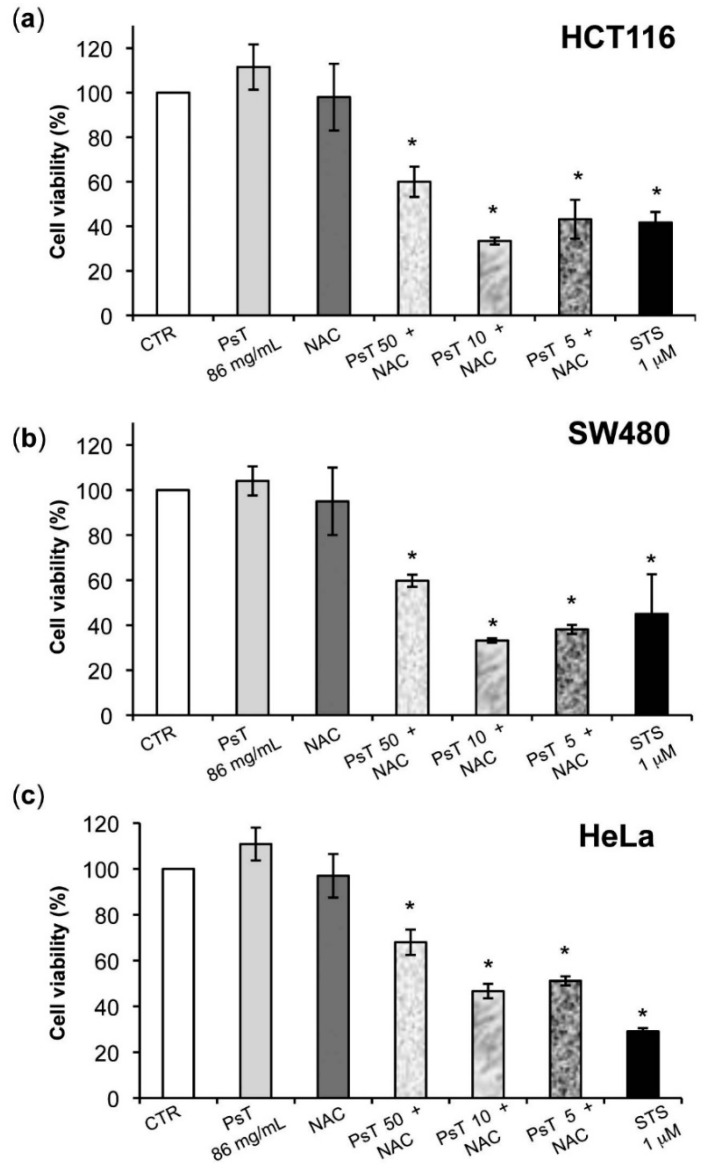

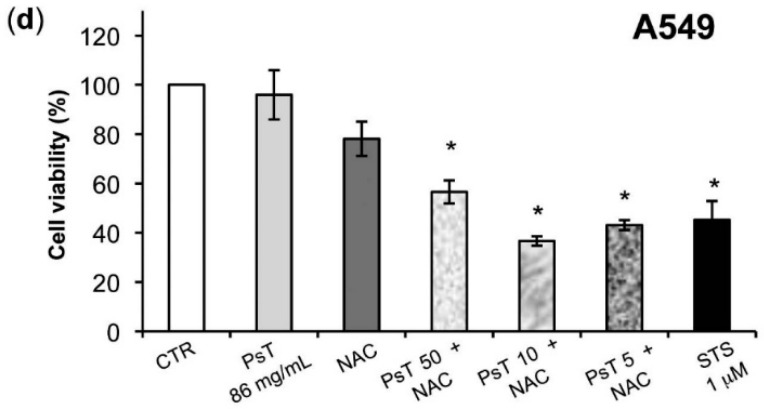
Effect of (*Prunus spinosa* Trigno ecotype plus Nutraceutical Activator Complex, PsT + NAC)^®^ combined treatments on different cell lines. HCT116 (**a**); SW480 (**b**); HeLa (**c**) and A549 (**d**) cells were treated with NAC alone, PsT 86 mg/mL, (PsT 50 mg/mL + NAC)^®^, (PsT 10 mg/mL + NAC)^®^, (PsT 5 mg/mL + NAC)^®^ for 24 h; Staurosporine (STS, 1 μM) was used as a positive control. Results showed that combined treatments were effective on all cell lines. Cell viability was assessed by 3-[4,5-dimethylthiazol-2-yl]-2,5-diphenyltetrazolium bromide (MTT) assay, performed for six independent experiments. One-way Analysis of Variance (ANOVA) was applied. * = significant differences compared to control cells (*p* < 0.05).

The effect of (PsT + NAC)^®^ on the HCT116, SW480, HeLa and A549 cell lines’ vitality was investigated by 3-[4,5-dimethylthiazol-2-yl]-2,5-diphenyltetrazolium bromide (MTT) (Figure 1a–d). MTT is able to assess the damage to the inner mitochondrial membrane caused by drugs or other toxicants. Treatment with NAC alone did not affect the vitality of the above tumor cells. The histograms a–d of Figure 1 show that PsT 86 mg/mL did not modify the cell line survival of HCT116, SW480, HeLa and A549.

As shown, the efficacy of (PsT + NAC)^®^ was proven in all analyzed cancer cells (*p* < 0.05). The MTT data show that treatment with (PsT 10 mg/mL + NAC)^®^ reduced tumor cell metabolic activity when compared to NAC or PsT alone (*p* < 0.001).

Post hoc analysis maintained differences (*p* < 0.05) between the control cells and all treatments for SW480. For the HCT116 cell line, differences (*p* < 0.001) emerged for control cells compared to (PsT 50 mg/mL + NAC)^®^, (PsT 10 mg/mL + NAC)^®^, and STS 1 µM. For the HeLa cell line, differences (*p* < 0.05) were found for control cells with respect to (PsT 50 mg/mL + NAC)^®^, (PsT 10 mg/mL + NAC)^®^, and STS 1 µM. For the A549 cell line, differences (*p* ≤ 0.01) emerged for control cells compared to (PsT 50 mg/mL + NAC)^®^, (PsT 10 mg/mL + NAC)^®^, and STS 1 µM were found.

**Figure 2:**
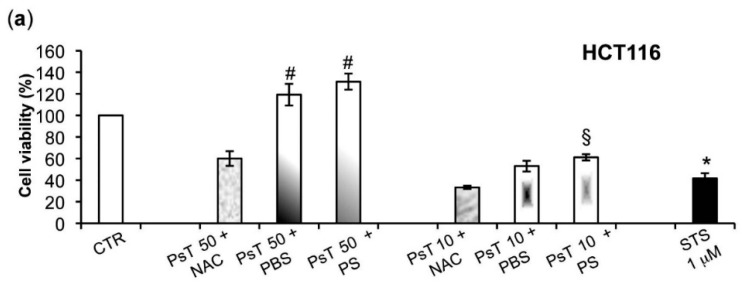

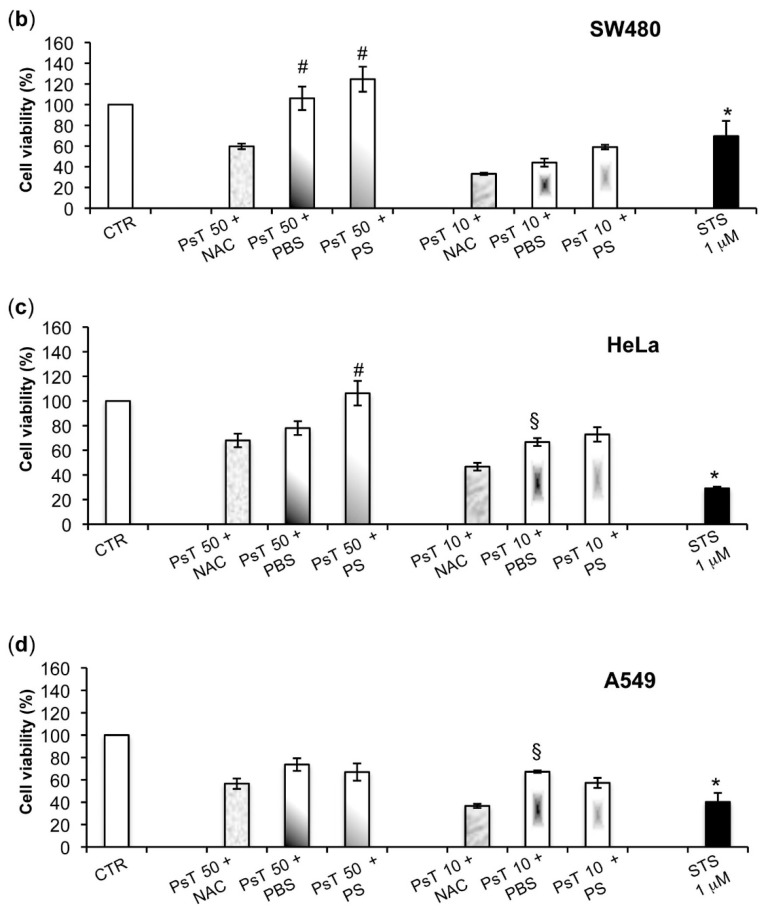
Cytotoxic effect determination of PsT 50 mg/mL and PsT 10 mg/mL diluted with NAC, phosphate buffer saline (PBS) or physiological solution (PS) vehicles. HCT116. (**a**) SW480 (**b**) HeLa (**c**) A549 (**d**) cells were treated for 24 h and cell viability was evaluated using an MTT test. Staurosporine (STS, 1 μM) was used as positive control. Results showed that PsT in association with NAC was effective in all examined cell lines. Cell viability was assessed by MTT assay, performed for six independent experiments. One-way ANOVA was applied. # = significant difference compared to cells treated with (PsT 50 mg/mL + NAC)^®^, *p* < 0.05; § = significant difference compared to cells treated with (PsT 10 mg/mL + NAC)^®^, *p* < 0.05; * = significant differences compared to control cells (*p* < 0.05).

Furthermore, to show that only the NAC vehicle, when combined with the *Prunus* extract, was responsible for the cytotoxic effect, we also used phosphate buffer solution (PBS) or physiological solution (PS) as alternate vehicles for PsT (Figure 2).

Figure 2a–d show that cell treatments with PsT 50 or PsT 10 mg/mL + PBS or physiological solution (PS), induced an increase of cancer cell survival compared to (PsT + NAC)^®^.

Both the HCT116 and SW480 cells lines recovered vitality of about 100% after PsT 50 + PBS or PS treatments; this was about 40–60% for PsT 10 + PBS or PS treatments (*p* < 0.05) (Figure 2a,b).

For the HeLa and A549 cells lines the vitality recovery was about 70% after PsT 50 or P10 + PBS or PS treatments (Figure 2c,d). PBS or PS alone did not change the cell survival (data not shown). These results show that the treatment with (PsT + NAC)^®^ is an effective combination against the tumor cells.

### 2.3. (PsT + NAC)^®^ Treatment-Induced Mitochondrial Membrane Depolarization on Colon Carcinoma Cells

After having verified that the combined treatment (PsT + NAC)^®^ was able to reduce the viability of numerous cancer cells, we deepened the action mechanism on two colon cancer lines (HCT116 and SW480). That investigation was possible as the use of *Prunus spinosa* extract had already been authorized by the Italian Health Authority to be sold as a diet supplement.

**Figure 3:**
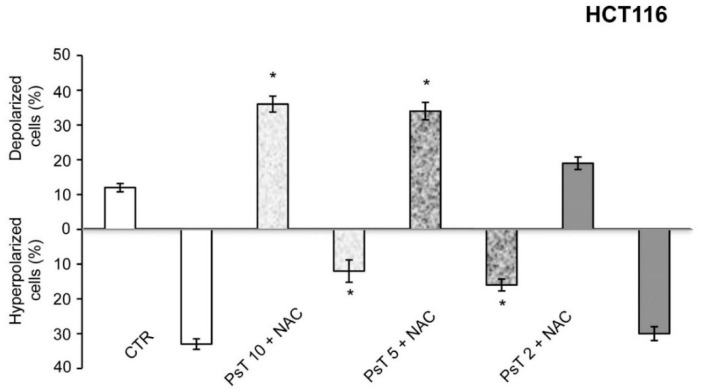
Effect of (PsT + NAC)^®^ treatment on mitochondria. Evaluation of mitochondrial membrane potential of HCT116 cells with tetramethylrhodamine methyl ester (TMRM) probe after PsT 10 mg/mL, PsT 5 mg/mL, PsT 2 mg/mL + NAC for 24 h. All combined treatments showed a dose-related mitochondrial depolarization. One-way ANOVA was applied; * = significant difference compared to control cells.

The use of flow cytometric analysis on mitochondrial membrane potential, performed after (PsT + NAC)^®^ treatment of HCT116 and SW480 cells (data not shown), demonstrated that the percentage of cells with depolarized mitochondria increases in a dose-dependent manner, whereas the percentage of cells with hyperpolarized mitochondria decrease (Figure 3). These results confirm the hypothesis, previously demonstrated with MTT tests, that the cytotoxic effect induced by (PsT + NAC)^®^ treatment is due to the unbalance of the electric potential between the inner and outer mitochondrial membrane.

### 2.4. The Effect of the Treatment with a Lower Concentration of (PsT + NAC)^®^ on the Clonogenic Survival of HCT116 and SW480 Cells

**Figure 4:**
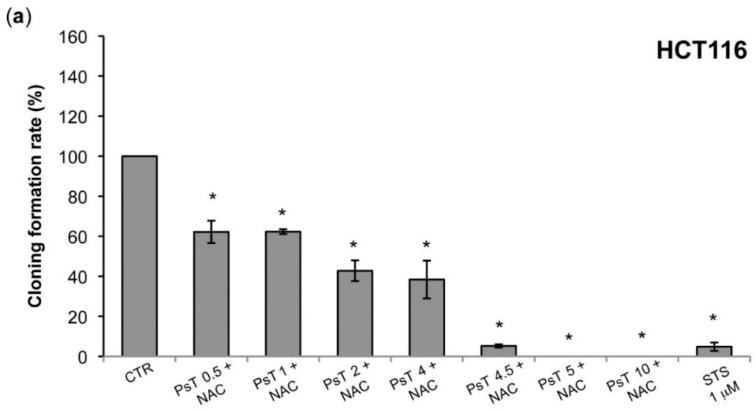

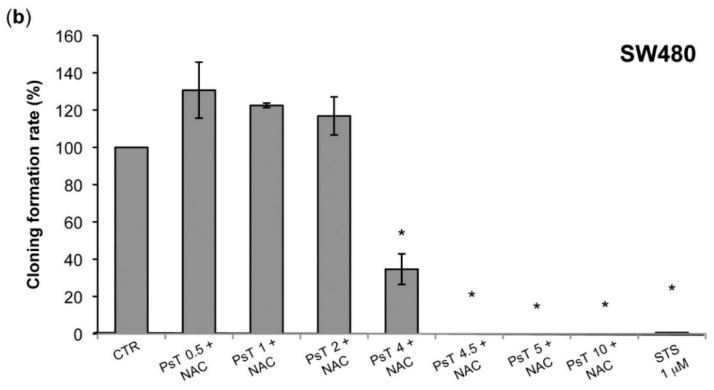
Clonogenic effect evaluated on HCT116 and SW480 cells after (PsT 0.5–10 mg/mL + NAC)^®^ treatments for 24 h. Staurosporine (STS, 1 μM) was used as positive control. All experiments were performed at least three times. One-way ANOVA was applied. * = significant difference compared to control cells *p* < 0.001.

The aim of this investigation was to assess the effect of (PsT + NAC)^®^ on the survival of HCT116 and SW480 cells at concentrations below 10 mg/mL (Figure 4).

The clonogenic survival experiments on HCT116 cells (Figure 4a) showed that at a dose of (PsT 0.5 mg/mL + NAC)^®^ survival was already effective (60%), rapidly decreasing to 10% at (PsT 4.5 mg/mL + NAC)^®^ and reaching a null value up to (PsT 10 mg/mL + NAC)^®^. In the case of SW480 cells treated with (PsT from 0.5 mg/mL to 2 mg/mL + NAC)^®^, the cell survival was 100%. The SW480 cells instead showed resistance at low doses. This behavior is attributed to different genotype expression between the two colon cancer cell lines (Figure 4b).

### 2.5. The (PsT + NAC)^®^ Combined Treatment Induces Apoptosis in Colon Cancer Cells

**Figure 5:**
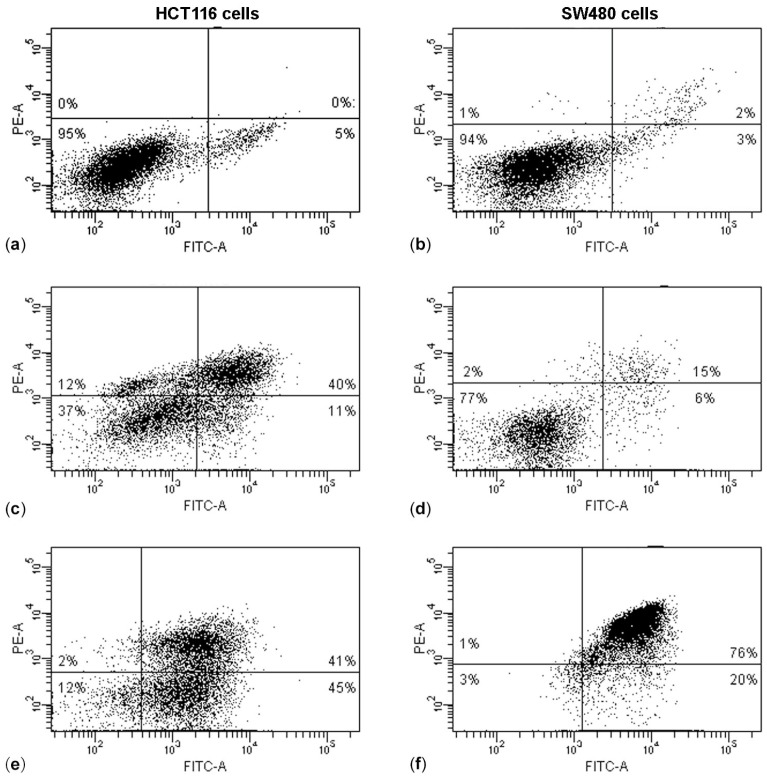
Apoptotic induction on HCT116 and SW480 cells performed by Annexin V-fluorescein isothiocyanate (FITC)/Propidium iodide (PI) test. HCT116 control cells (**a**) treated with (PsT 5 mg/mL + NAC)^®^; (**c**) (PsT 10 mg/mL + NAC)^®^; (**e**) for 24 h. SW480 control cells (**b**) treated with (PsT 5 mg/mL + NAC)^®^; (**d**) (PsT 10 mg/mL + NAC)^®^, (**f**) for 24 h. Dot plots are representative of three independent experiments.

Simultaneous use in all experiments of Staurosporine as a positive control seems to indicate that cytotoxicity is due to apoptotic cell death [13]. To cross-check this fact, an Annexin V-fluorescein isothiocyanate (FITC)/Propidium iodide (PI) assay was performed by flow cytometry on colon carcinoma cells after (PsT + NAC)^®^ treatment (Figure 5). The dot plots in Figure 5 reveal that HCT116 cells, after 24 h of treatment with either (PsT 5 mg/mL + NAC)^®^ (Figure 5c) or (PsT 10 mg/mL + NAC)^®^ (Figure 5e), undergo apoptosis (51% or 86% apoptotic cells fraction, respectively, against 5.0% of control cells).

On SW480 cells, we observed, instead, a slight increase in the apoptotic fraction (21%) at treatment for 24 h with (PsT 5 mg/mL + NAC mg/mL)^®^ and a further increase to 96% with (PsT 10 mg/mL + NAC)^®^.

**Figure 6:**
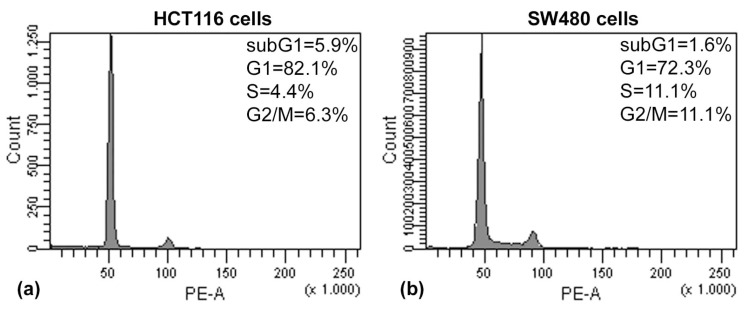

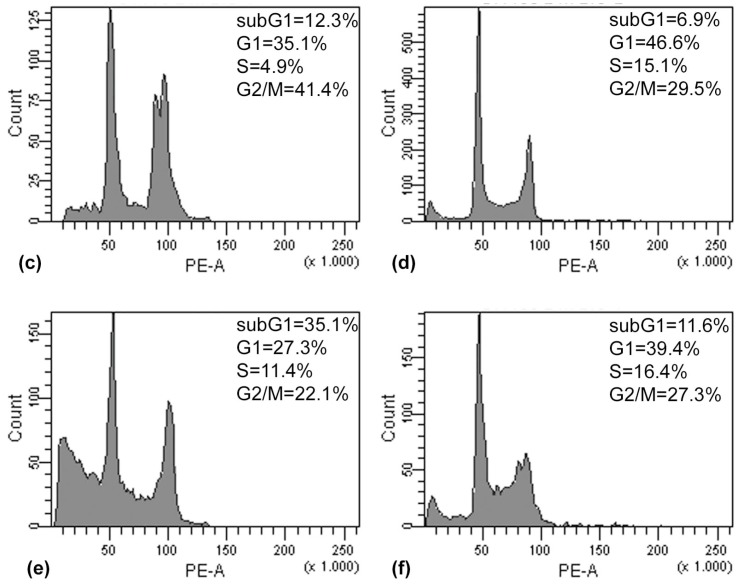
Cell cycle analysis on HCT116 and SW480 cells by DNA labeling with Propidium Iodide (PI). HCT116 control cells (**a**) treated with (PsT 5 mg/mL + NAC)^®^; (**c**) (PsT 10 mg/mL + NAC)^®^; (**e**) for 24 h. SW480 control cells (**b**) treated with (PsT 5 mg/mL + NAC)^®^; (**d**) (PsT 10 mg/mL + NAC)^®^; (**f**) for 24 h. Histograms are representative of three independent experiments.

Moreover, a careful analysis of the cell cycle has been carried out by flow cytometry on colon carcinoma cells treated with (PsT + NAC)^®^. Cell cycle analysis of HCT116 and SW480 cells treated with (PsT + NAC)^®^ for 24 h showed a dose-dependent increase of sub G1 peak and cell uptake in the Gap 2/Mitosis phase (G2/M) (Figure 6).

We observed in the G2/M phase a significant increase after treatment with (PsT 5 mg/mL + NAC)^®^ on both cell lines: 41.4% for HCT116 cells (Figure 6c) and 29.5% for SW480 cells (Figure 6d).

After treatment of HCT116 cells with (PsT 5 mg/mL + NAC)^®^, the sub-G1 peak increased (12.3%, Figure 6c) in comparison with the control cells (5.9%, Figure 6a) and the G2/M phase raised up to 41.4% (Figure 6c) against 6.3% of the control cells (Figure 6a).

The same effect on the sub-G1 peak was verified on SW480 cells (6.9% for cells treated with (PsT 5 mg/mL + NAC)^®^ (Figure 6d) and 11.6% for cells treated with (PsT 10 mg/mL + NAC)^®^ (Figure 6f) in comparison with 1.6% of the control cells (Figure 6b). These results confirmed that (PsT + NAC)^®^ induced apoptotic cell death.

### 2.6. Effect of (PsT + NAC)^®^ on Normal Human Cells

**Figure 7:**
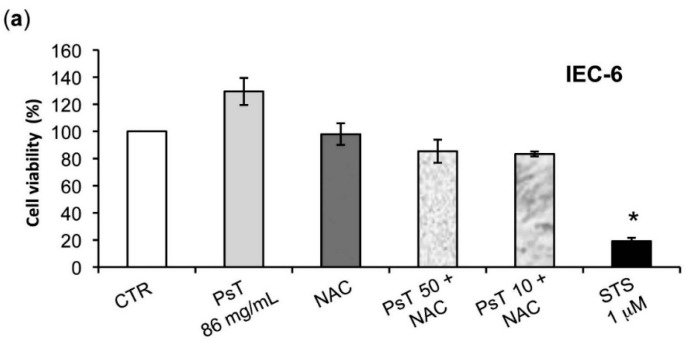

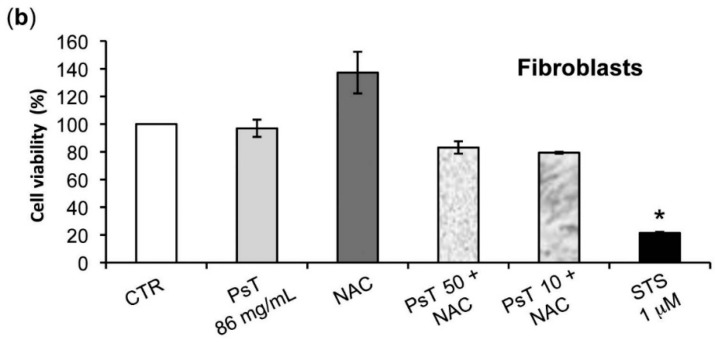
Effect of (PsT + NAC)^®^ on normal cells. (**a**) Viability of IEC-6 cells; (**b**) the viability of human gingival fibroblasts (**b**) was evaluated after treatment with NAC alone, PsT 86 mg/mL alone, (PsT 50 mg/mL + NAC)^®^, and (PsT 10 mg/mL + NAC)^®^ and Staurosporine (STS) as a positive apoptosis control, for 24 h. The results showed that the combined treatments did not affect normal cells. Cell viability was assessed by MTT assay, performed for six independent experiments. One-way ANOVA was applied. * = significant differences compared to control cells (*p* < 0.05).

The effects of NAC alone, of PsT 86 mg/mL, of (PsT 50 mg/mL + NAC)^®^, and of (PsT 10 mg/mL + NAC)^®^, in each case for 24 h, were also evaluated on normal cells (Figure 7). After treatment with (PsT 50 mg/mL + NAC)^®^, or (PsT 10 mg/mL + NAC)^®^, IEC-6 and fibroblasts, cell vitality did not change in comparison to NAC treatment alone (about 80% for both cell lines) (Figure 7a,b). These data show that the combined (PsT + NAC)^®^ treatments are highly effective on cancer cells but not on normal cells.

## 3. Discussion

The antioxidant activity of berries and drupes, like those of the *Prunus spinosa* Trigno ecotype, is well known also for their use in the field of foodstuffs [6,14]. The chemical composition has been identified and quantified by liquid chromatography coupled to mass spectrometry. The plant extract (PsT) is characterized by the presence of active compounds as phenolic acids, flavonoids and anthocyanins. In particular, higher quantities are found of the phenolic acid group (3-*O*-Caffeoylquinic and the 4-*O*-Caffeoylquinic acids), the flavonoid group (quercetins and Kaempferol 3-*O*-rutinoside), and the anthocyanins group (cyanidins and peonidins) (Table 1). The extract has proven to be particularly effective for its high presence and special distribution of flavones, flavonols, phenolic acids and anthocyanins, all active components known for their antioxidant and antiproliferative activities [6,7]. Initially our study aimed at the assessment of the cytotoxic effects of this compound on histologically-different cell lines as the human colon, cervix and lung carcinoma. The analysis of the cellular vitality of tumor lines has shown that both *Prunus spinosa* and NAC “alone” do not show any toxicity to the above human cancer cells. However, when *Prunus spinosa* is diluted with NAC, there is a noticeable cytotoxicity effect in all cancer cells.

The cytotoxicity phenomenon observed may be ascribed to the induction of apoptosis, as the positive control used in all experiments has shown (Figure 1).

To verify whether or not the NAC is responsible for the observed cytotoxicity in the cancer cells, we experimented by using other vehicles, namely both PBS and PS, to dilute *Prunus spinosa* (PsT 86 mg/mL).

We found that the (PsT + NAC)^®^ combination effectively reduced tumor cell survival, and, conversely, that the PsT + PBS and PsT + PS, do not (Figure 2).

Our study continued using two genetically different human colon carcinoma lines (HCT116 and SW480). This decision was taken because the ingestion of flavonoid sugar moieties, which are cleaved from the phenolic backbone in the small intestine, are adsorbed here, while only a small part of ingested anthocyanins are absorbed at the small intestine level and large amounts of these latter compounds enter the colon, where they are de-glycosylated by gut microbiota [15].

The investigation of the anti-tumor effect of the flavonoids and anthocyanins that are found in high amounts in the *Prunus* food supplement on human colon carcinoma cells seemed to us very promising. The MTT test evaluation showed that mitochondria could be directly connected in the action mechanism of these complexes. The test involves chemical reactions with NAD (P) H-dependent cellular oxidoreductase enzymes, which provide the number of viable cells in the whole population. The result shows a reduction in the mitochondrial activity [16].

Cancer cells have a more hyperpolarized mitochondrial membrane potential (ΨIM) than normal cells; ΨIM in cancer cells is about 220 mV, whereas in normal cells it is about 140 mV [17]. Owing to the difference in polarization between normal and tumor cells, and since this compound has the characteristic of depolarizing mitochondrial membrane, it may be selectively effective on cancer cells.

To prove the correctness of our hypothesis, we quantified the percentage of cells with depolarized and hyperpolarized mitochondria by using cytofluorimetry quantitative analysis with (PsT + NAC)^®^. The result shows that it induces mitochondrial membrane depolarization of colon carcinoma cells (Figure 3) and hence that (PsT + NAC)^®^ has a targeted cytotoxic action.

Our results are strengthened by the presence of high amounts of active ingredient in flavonoids as shown in Table 1, where the drupe composition of PsT is reported. Therefore, C2=C3 double bonds/3-OH groups, in conjugation with the 4-oxo function of the C-ring in the flavonoid structure, favor the interaction of these compounds with the mitochondrial membrane, decreasing its fluidity either by inhibiting the respiratory chain of mitochondria or by causing uncoupling [18].

A clonogenic analysis was performed, which confirmed the cytotoxic effect, evaluated by the enzymatic test (Figure 1 and Figure 2). At 10 mg/mL the treatment was effective on both HCT116 and SW480 lines, while at a lower dose, from 0.5 mg/mL to 2 mg/mL, only the SW480 was resistant (Figure 4). Although these cell lines were both colorectal cancer, they have different epigenetic and genetic features [19]. In particular, the SW480 cell line was characterized by TP53 mutations, which made it resistant to apoptosis induction and, consequently, delayed the apoptotic effect of combined treatment in comparison with HCT116 cells.

The cytotoxic activity of (PsT + NAC)^®^ might be related to its peculiar constituents and the NAC vehicle, that might together increase the bioavailability of some compounds, such as quercetin [20], present in large amounts in our ecotype (Table 1).

Unfortunately, the clinical use of pure quercetin is limited due to its low water solubility and instability in physiological media that lead to low intestinal absorption [21]. Recent studies have shown that a high amount of “found-food quercetin” can be easily absorbed from the intestine and subsequently converted into the active constituents [22], showing cancer prevention and therapeutic effects in vitro as well as in vivo [21].

The results of the Annexin V/PI assay and cell cycle analysis, shown in Figure 5 and Figure 6, demonstrated that (PsT + NAC)^®^ combined treatment-induced apoptotic cell death in HCT116 and SW480 cells. However, HCT116 cells were more sensitive than SW480 cells, as demonstrated by the evident arrest of the G2/M phase after (PsT 5 mg/mL + NAC)^®^ combination, and by the remarkable subG1 peak after (PsT 10 mg/mL + NAC)^®^ combination.

The analysis of *Prunus spinosa* + NAC treatments on normal human cell lines showed that, at the same concentrations, this compound is not cytotoxic, giving a greater importance to the obtained results (Figure 7).

In conclusion, the diversity of the bioactive compounds of the PsT extract show that it is a good source of phytochemicals, and until now has been considered a diet supplement only, due to its healthy nutritional profile. Its great efficacy as an antiproliferative compound on cancer cell lines, could be attributed to the special nature and distribution in the plant complex characterized by the enrichment in flavones, flavonols, phenolic acids and anthocyanins. However, it was found that PsT extract alone did not modify the survival of cancer cells. Only the PsT extract in combination with NAC reduced cell viability significantly, also at low doses.

Further in vivo animal model studies are in progress to investigate the use of (PsT + NAC)^®^ together with proven chemotherapeutic agents, aiming at the improvement of efficacy, to reduce the drugs doses and their undesired collateral effects as a short-term overall benefit.

To go forward from in vivo to clinic, we will have to identify which of the (PsT + NAC)^®^ chemical components are the factors triggering cytotoxicity and apoptosis in cancerous cells, as this is an important step for the future synthesis of the active principles or molecules to perform specific pharmacokinetic and pharmacodynamic studies.

We hope that this will lead from a “food supplement” to a real anticancer drug, as has happened with other “plant derived” chemotherapy.

## 4. Materials and Methods

### 4.1. Plant Material

Fully mature blackthorn fruits of the *Prunus spinosa* Trigno ecotype (PsT) were collected in late October 2014 by hand picking in the district of Bagnoli del Trigno, which is about 35 km north east of Isernia (Molise Region, Italy, latitude 41°42′ N, longitude 14°27′ E, altitude 650 m a.s.l.). The Molise region is positioned on the eastern side of the Apennines watershed, and has the typical Mediterranean climate of south-central Italy. The area has an average annual rainfall of 850 mm, and a mean annual temperature of 12.6 °C. This territory includes an area with a low population density, reduced road traffic, as well as low levels of photochemical smog and fine particles. The morphological key characteristics used for the plant identification were taken from Flora d’Italia [23]. The fruits were kept in cooled bags and then stored in a deep-freezer at −20 °C for subsequent analysis. Three samples were used and all the assays were carried out in triplicate. The results were given by the average values and the errors by the standard deviation (SD).

### 4.2. Plant Extraction and HPLC-DAD–ESI/MS Analysis of Phenolic Acids and Flavone/Ols

HPLC separation of the phenolic acids and flavone/ols extract was performed according to Guimarães and colleagues [24]. Briefly, each sample of dried and ground fruit was extracted with methanol: water 80:20 (*v*/*v*) at room temperature, 150 rpm, for 1 h. The extract was filtered through Whatman No. 4 paper. The residue was then re-extracted twice with additional 30 mL portions of methanol: water 80:20 (*v*/*v*). The combined extracts were evaporated at 35 °C (rotary evaporator Büchi R-210 (Marshall Scientific, Hampton, VA, USA,) to remove methanol. For purification, the aqueous phase was deposited onto a C-18 SepPak^®^-Vac 3 cc cartridge (Phenomenex, Torrance, CA, USA).

The extracts were analyzed using a Hewlett–Packard 1100 chromatograph (Agilent Technologies, San Diego, CA, USA) with a diode array detector (DAD) coupled to an HP Chem Station (rev. A.05.04) data-processing station. A Waters Spherisorb S3 ODS-2 C18, 3 µm (4.6 × 150 mm) column thermostat set at 35 °C was used. The solvents used were: (A) 0.1% formic acid in water; (B) acetonitrile. Double online detection was carried out in the DAD using 280 and 370 nm as the preferred wavelengths and in a mass spectrometer (MS) connected to the HPLC system via the DAD cell outlet.

MS detection was performed in an API 3200 Qtrap (Applied Biosystems, Darmstadt, Germany) equipped with an ESI source and a triple quadrupole ion trap mass analyzer that was controlled by the Analyst 5.1 software (Merck, Saint Louis, MO, USA). The MS detector was programmed for recording in two consecutive modes: enhanced MS (EMS) and enhanced product ion (EPI) analysis. EMS was employed to show the full spectra, so as to obtain an overview of all of the ions in each sample.

The phenolic compounds in the samples were characterized according to their UV and mass spectra and retention times compared to available standards. For the quantitative analysis of phenolic compounds, a five-level calibration curve was obtained by the injection of known concentrations (2.5–100 µg/mL) of different standard compounds: caffeic acid, chlorogenic acid, gallic acid, isorhamnetin 3-*O*-glucoside, isorhamnetin 3-*O*-rutinoside, kaempferol 3-*O*-glucoside, kaempferol 3-*O*-rutinoside, quercetin 3-*O*-glucoside and quercetin 3-*O*-rutinoside. The results were expressed in mg per 100 g of dry weight (dw).

### 4.3. Plant Extraction and HPLC-DAD–ESI/MS Analysis of Anthocyanins

The analysis of the anthocyanins extract was performed according to Guimarães and colleagues [24]. Briefly, they were extracted with methanol containing 0.5% trifluoroacetic acid (TFA) and filtered through a Whatman No. 4 paper. The residue was then re-extracted twice with additional 30 mL portions of 0.5% TFA in methanol. The combined extracts were evaporated at 35 °C to remove the methanol, and redissolved in water. For purification, the extract solution was deposited onto a C-18 SepPak^®^- Vac 3 cc cartridge (Phenomenex).

The extracts were analyzed using the HPLC system and separation was achieved on an AQUA^®^-(Phenomenex) reverse phase C18 column (5 µm, 150 × 4.6 mm i.d.) with the thermostat set at 35 °C. The solvents used were: (A) 0.1% TFA in water; and (B) 100% acetonitrile. Double detection was carried out by DAD, using 520 nm as the preferred wavelength, and MS, using the same equipment described above. The EMS and ESI methods were used for the acquisition of the full spectra and fragmentation patterns of the precursor ions, respectively.

The anthocyanins present in the samples were characterized according to their UV and mass spectra and retention times, and comparison with authentic standards. For quantitative analysis, a five-level calibration curve was obtained by the injection of known concentrations (50–0.25 µg/mL) of different standard compounds: cyaniding 3-*O*-glucoside and peonidin 3-*O*-glucoside. The results were expressed in µg per 100 g of dry weight (dw).

### 4.4. Cell Cultures

Human colorectal carcinoma cells (HCT116), human colorectal adenocarcinoma cells (SW480), human cervical cancer cells (HeLa), human bronchoalveolar adenocarcinoma cells (A549), human gingival fibroblasts, and rat intestinal epithelial cells (IEC-6) were provided by the American Type Culture Collection (ATCC, Manassas, VA, USA) and used according to Meschini et al. [12].

### 4.5. Cell Treatments

The PsT extraction was performed by macerating the vegetable material in a water/alcohol solvent (60° of alcohol) for varying periods, from a few hours to several days. The drying process was performed using the conventional methods for evaporation under reduced pressure, spray drying, or lyophilization. The dry weight of PsT used for the cell culture treatments ranged from 86 mg to 0.017 mg of the total. Cells were treated with PsT hydroalcoholic solution (86 mg/mL), or with different solutions (50, 10, 5, 4.5, 4, 2, 1, 0.5, 0.1 mg/mL) obtained after the progressive dilution of PsT 86 mg/mL with a complex blend of amino acids, vitamins and minerals, called nutraceutical activator complex (NAC), for 24 h [12].

The experiments were also performed treating cells with PsT diluted with other vehicles, such as phosphate saline buffer (PBS, Sigma-Aldrich, Saint Louis, MO, USA) or 0.9% physiological solution (PS), to demonstrate that the (PsT + NAC)^®^ combined action was the most effective against tumor cells.

To perform a positive control of apoptosis induction, the cells were treated with Staurosporine (STS, 1 µM, Sigma-Aldrich, Saint Louis, MO, USA) for 24 h [13].

### 4.6. MTT Assay

Cell viability was assessed by (3-[4,5-dimethylthiazol-2-yl]-2,5-diphenyltetrazolium bromide) MTT assay (Sigma Aldrich, Saint Louis, MO, USA).

After removing the cell medium, untreated and (PsT + NAC)^®^ treated cells were washed with PBS and incubated with 0.5 mg/mL MTT solution for 2 h at 37 °C. After removing the MTT solution, the samples were lysed by 100 μL DMSO, and analyzed by a microplate reader (Bio-Rad, Hercules, CA, USA) at 570 nm. Cell viability (%) was calculated as follows: (absorbance mean value of the treated sample/absorbance mean value of the control sample) × 100 [25].

### 4.7. Detection of Mitochondrial Membrane Potential

Cationic fluorescent probe tetramethylrhodamine methyl ester (TMRM, Molecular Probes Inc., Eugene, OR, USA) was used to monitor the loss of mitochondrial membrane potential [26]. Untreated and treated HCT 116 and SW480 cell suspensions were stained with TMRM solution (25 µg/mL) for 10 min at 37 °C and analyzed by flow cytometry.

### 4.8. Cloning Efficacy Assay

Untreated and treated HCT116 and SW480 cells were detached and plated (1 × 10^3^) per 60 mm tissue culture dish and allowed to grow in culture medium for 15 days. After growth, cell colonies were fixed with 95% ethanol for 15 min, and stained with a methylene blue solution in 80% ethanol for 2 h. Only colonies composed of more than 50 cells were evaluated. The cloning formation rate (%) was calculated by dividing the number of colonies of treated cells, and the number of colonies of untreated cells.

### 4.9. Annexin V-FITC/PI Assay

Annexin V-fluorescein isothiocyanate (FITC)/Propidium iodide (PI) staining was used to investigate cell death induced by the combined treatment PsT^®^ + NAC. After treatment for 24 h, cells were processed using an Annexin V-FITC/PI apoptosis detection kit (eBioscence, London, UK). They were detached, centrifuged and re-suspended in binding buffer 1X. Cell suspensions were then incubated with 5 µL of Annexin V-FITC solution for 15 min. After washing with binding buffer 1X, cells were incubated with 5 µL of PI and immediately analyzed by flow cytometer.

### 4.10. Cell Cycle Analysis

Untreated and treated HCT 116 and SW480 cell pellets were fixed in 70% ethanol in PBS at 4 °C for 1 h, washed twice and then re-suspended in PBS containing 100 µg/mL ribonuclease (RNAse, Sigma-Aldrich, St. Louis, MO, USA). Cellular DNA was labelled with 40 µg/mL PI in PBS and stored at 37 °C, for at least 30 min. After this, incubation cells were analyzed by flow cytometry.

### 4.11. Flow Cytometric Analyses

Flow cytometric analyses were carried out by a BD LSR II flow cytometer (Becton, Dickinson & Company, Franklin Lakes, NJ, USA) equipped with a 15 mW, 488 nm, air-cooled argon ion laser and a Kimmon HeCd 325 nm laser. At least 10,000 events were acquired in lin or log mode. The percentage of depolarized/hyperpolarized cells, quantitative analysis of apoptosis, and cell cycle distribution were performed using FACS Diva Software (Becton, Dickinson & Company).

### 4.12. Statistical Analyses

The distribution of each measurement was examined for the assumption of normality with the Shapiro–Wilk test. One-way Analysis of Variance (ANOVA) was applied to detect differences between the control and treatments. Bonferroni post hoc analysis was applied to reveal differences between all treated samples in each cell line. The alpha level was set at *p* < 0.05.

## Figures and Tables

**Table 1:** Phenolic acids, flavones/ols and anthocyanins of fully mature blackthorn fruits.

Compounds	Relative Values (mg/100 g or µg/100 g Dry Weight)
3-*O*-Caffeoylquinic acid	26.31 ± 0.26
3-p-Coumaroylquinic acid	0.77 ± 0.02
4-*O*-Caffeoylquinic acid	8.97 ± 0.08
3-*O*-Feruloylquinic acid	2.31 ± 0.01
**Total phenolic acids**	**38.36 ± 0.19 mg/100 g**
Caffeoyl hexoside	0.99 ± 0.01
Quercetin pentosylhexoside	1.58 ± 0.02
Quercetin rhamnosylhexoside	2.01 ± 0.01
Quercetin 3-Orutinoside	21.98 ± 0.09
Quercetin 3-Oglucoside	8.92± 0.04
Quercetin hexoside	4.92 ± 0.03
Kaempferol 3-Orutinoside	6.22 ± 0.02
Quercetin hexosylrhamnoside	7.51 ± 0.05
Quercetin pentoside	7.99 ± 0.06
Quercetin rhamnoside	1.26 ± 0.01
Quercetin acetylrutinoside	1.24 ± 0.02
**Total flavone/ols**	**64.62 ± 0.58 mg/100 g**
Cyanidin 3-*O*-glucoside	24.25 ± 0.19
Cyanidin 3-*O*-rutinoside	42.31 ± 0.35
Peonidin 3-*O*-glucoside	13.91 ± 0.11
Peonidin 3-*O*-rutinoside	15.53 ± 0.09
Cyanidin 3-*O*-pentoside	1.63 ± 0.01
Peonidin 3-*O*-acetylglucoside	0.99 ± 0.01
**Total anthocyanins**	**0.63 μg/100 g**

## References

[1] Mauricio S.F., Ribeiro H.S., Correia M.I. (2016). Nutritional status parameters as risk factors for mortality in cancer patients. Nutr. Cancer.

[2] Hussain S.A., Sulaiman A.A., Balch C., Chauhan H., Alhadidi Q.M., Tivari A.K. (2016). Natural polyphenols in cancer chemoresistance. Nutr. Cancer.

[3] Chen A.Y., Chen Y.C. (2013). A review of the dietary flavonoid, kaempferol on human health and cancer chemoprevention. Food Chem..

[4] Newman D.J., Cragg G.M. (2007). Natural products as sources of new drugs over the last 25 years. J. Nat. Prod..

[5] Calvo M.I., Cavero R.Y. (2014). Medicinal plant used for cardiovascolar deseases in Navarra and their validation in official sources. J. Ethnopharmacol..

[6] Szajdek A., Borowska E.J. (2008). Bioactive compounds and health-promoting properties of berry fruits: A review. Plant Foods Hum. Nutr..

[7] Roleira F.M., Tavares-da-Silva E.I., Varela C.L., Costa S.C., Silva T. (2015). Plant derived and dietary phenolic antioxidants: Anticancer properties. Food Chem..

[8] Scalbert A., Williamson G. (2000). Dietary intake and bioavailability of polyphenols. J. Nutr..

[9] Busch C., Burkard M., Leischner C., Lauer U.M., Frank J., Venturelli S. (2015). Epigenetic activities of flavonoids in the prevention and treatment of cancer. Clin. Epigenetics.

[10] Gilbert E.R., Liu D. (2010). Flavonoids influence epigenetic-modifying enzyme activity: Structure—Function relationships and the therapeutic potential for cancer. Curr. Med. Chem..

[11] Kong J.M., Chia L.S., Goh N.K., Chia T.F., Brouillard R. (2003). Analysis and biological activities of anthocyanins. Phytochemistry.

[12] Meschini S., Mastrodonato F. (2015). Estratti di Prunus spinosa ad Attività Antitumorale. Italian Patent.

[13] Belmokhtar C.A., Hillion J., Ségal-Bendirdjian E. (2001). Staurosporine induces apoptosis through both caspase-dependent and caspase-independent mechanisms. Oncogene.

[14] Fraternale D., Giampieri L., Bucchini A., Sestili P., Paolillo M., Ricci D. (2009). *Prunus spinosa* fresh fruit juice: Antioxidant activity in cell-free and cellular systems. Nat. Prod. Commun..

[15] Marìn L., Miguélez E.M., Villar C.J., Lombò F. (2015). Bioavailability of dietary polyphenols and gut microbiota metabolism: Antimicrobial properties. Biomed. Res. Intern..

[16] Van Meerloo J., Kaspers G.J., Cloos J. (2011). Cell sensitivity assays: The MTT assay. Methods Mol. Biol..

[17] Modica-Napolitano J.S., Kulawiec M., Singh K.K. (2007). Mitochondria and human cancer. Curr. Mol. Med..

[18] Dorta D.J., Pigoso A.A., Mingatto F.E., Rodrigues T., Prado I.M., Helena A.F., Uyemura S.A., Santos A.C., Curti C. (2005). The interaction of flavonoids with mitochondria: Effects on energetic processes. Chem. Biol. Interact..

[19] Ahmed D., Eide P.W., Eilertsen I.A., Danielsen S.A., Eknaes M., Hektoen M., Lind G.E., Lothe R.A. (2013). Epigenetic and genetic features of 24 colon cancer cell lines. Oncogenesis.

[20] Appleton J. (2010). Evaluating the bioavailability of isoquercetin. Nat. Med. J..

[21] Khan F., Niaz K., Maqbool F., Ismail Hassan F., Abdollahi M., Naguipalla Venkata K.C., Nabavi S.M., Bishayee A. (2016). Molecular targets underlying the anticancer effects of Quercetin: An update. Nutrients.

[22] Murota K., Shimizu S., Miyamoto S., Izumi T., Obata A., Khikuci M., Terao J. (2002). Unique uptake and transport of isoflavone aglycones by human intestinal Caco-2 cells: Comparison of isoflavonoids and flavonoids. J. Nutr..

[23] Pignatti S. (2002). Flora d'Italia.

[24] Guimarães R., Barros L., Dueñas M., Carvalho A.M., Queiroz M.J., Santos-Buelga C., Ferreira I.C. (2013). Characterisation of phenolic compounds in wild fruits from Northeastern Portugal. Food Chem..

[25] Berridge M.V., Herst P.M., Tan A.S. (2005). Tetrazolium dyes as tools in cell biology: New insights into their cellular reduction. Biotechnol. Annu. Rev..

[26] Farkas D.L., Wei M., Febbroriello P., Carson J.H., Loew L.M. (1989). Simultaneous imaging of cell and mitochondrial membrane potentials. Biophys. J..

